# New Understanding of the Relevant Role of LINE-1 Retrotransposition in Human Disease and Immune Modulation

**DOI:** 10.3389/fcell.2020.00657

**Published:** 2020-08-07

**Authors:** Xiao Zhang, Rui Zhang, Jinpu Yu

**Affiliations:** ^1^Cancer Molecular Diagnostics Core, Tianjin Medical University Cancer Institute & Hospital, National Clinical Research Center of Caner, Key Laboratory of Cancer Prevention and Therapy, Key Laboratory of Cancer Immunology and Biotherapy, Tianjin, China; ^2^Tianjin’s Clinical Research Center for Cancer, Tianjin, China

**Keywords:** retrotransposons, LINE-1, regulatory mechanisms, cancer, immune, inhibitor

## Abstract

Long interspersed nuclear element-1 (LINE-1) retrotransposition is a major hallmark of cancer accompanied by global chromosomal instability, genomic instability, and genetic heterogeneity and has become one indicator for the occurrence, development, and poor prognosis of many diseases. LINE-1 also modulates the immune system and affects the immune microenvironment in a variety of ways. Aberrant expression of LINE-1 retrotransposon can provide strong stimuli for an innate immune response, activate the immune system, and induce autoimmunity and inflammation. Therefore, inhibition the activity of LINE-1 has become a potential treatment strategy for various diseases. In this review, we discussed the components and regulatory mechanisms involved with LINE-1, its correlations with disease and immunity, and multiple inhibitors of LINE-1, providing a new understanding of LINE-1.

## Introduction

Long interspersed nuclear elements (LINEs) are the only autonomous and active retrotransposons, which include LINE-1, LINE-2, and LINE-3 (Cordaux and Batzer, 2009; de Koning et al., 2011). Also, 5–6% of LINE-2 and LINE-3 sequences in the human genome are as a truncated molecular fossil (Doxiadis et al., 2012; Ardeljan et al., 2017). LINE-1 retrotransposons are one of the most abundant and effective classes of mobile DNAs that account for 17% of the human genome (Lander et al., 2001; Hancks and Kazazian, 2016). Full-length LINE-1 is 6.0–7.0 kb and contains a 5′-untranslated region (5′-UTR) (Swergold, 1990), two open reading frames (ORF1 and ORF2), and a 3′-UTR punctuated with a poly-A tract (Babushok and Kazazian, 2007; Beck et al., 2011). Denli et al. (2015) revealed a new open reading frame, ORF0. It is located in the 5′-UTR of the LINE-1 transcript and on the strand opposite of the ORF1 and ORF2 structural genes. Antisense promotor (ASP) can initiate fusion transcripts and regulate ORF0 to enhance LINE-1 mobility (Roman-Gomez et al., 2005; Weber et al., 2010; Criscione et al., 2016).

Both ORFs are required for LINE-1 retrotransposition process. ORF1 encodes an RNA-binding protein named ORF1P that has nucleic acid chaperone activity, and ORF2 encodes a protein named ORF2P that has endonuclease and reverse-transcriptase activities (Mathias et al., 1991; Feng et al., 1996). The first step occurs when RNA polymerase II binds to the 5′-UTR promoter region of LINE-1 and mediates the transcription of full-length mRNA of LINE-1 (Lavie et al., 2004). The LINE-1 mRNA is exported to the cytoplasm where ORF1 and ORF2 are translated and combined to form a ribonucleoprotein (RNP) particle. The RNP is then incorporated into the nucleus, and the ORF2P endonuclease in the RNP identifies and cuts specific sequences on the bottom DNA strand at the consensus site 3-′AA/TTTT−5′. Subsequently, the free 3′ hydroxyl generated at the fracture is utilized by the ORF2P and LINE-1 mRNA in the RNP is used as the template for reverse transcription to produce the complementary DNA of the LINE-1 gene (Wei et al., 2001; Hancks and Kazazian, 2016; Wang and Jordan, 2018). The distribution of LINE-1 in the human genome is selective. LINE-1 endonuclease activity and DNA replication determine LINE-1 insertion preference (Flasch et al., 2019). For example, LINE-1 preferentially inserts into nucleosome-depleted DNA primarily as a result of its AT-rich sequences (Sultana et al., 2019). The direction of the DNA replication fork affects LINE-1 insertion preference because the cleaved strand is usually the lagging strand template.

LINE-1 elements play a crucial role in the course of species formation and evolution. On one hand, de-repressed LINE-1 functions as a driver of many diseases and even a diagnostic marker for some diseases (Pedersen and Zisoulis, 2016). On the other, it can affect the developmental processes and influence the behavior by generating multiple gene products and causing variable deleterious effects on the structure of the host genome through new insertions, deletions, and recombinations (Garcia-Perez et al., 2016). LINE-1 RNA and protein overexpression is related to apoptosis, DNA damage and repair, cellular plasticity, and stress responses and can even promote tumor progression (Morrish et al., 2002, 2007; Belgnaoui et al., 2006; Sinibaldi-Vallebona et al., 2006). DNA damage caused by genome-wide or intersperse repetitive sequences hypomethylation can induce inflammatory microenvironment (Lindqvist et al., 2017; Teerawattanapong et al., 2019). Here, we reviewed the correlation between LINE-1 and disease as well as immune system, meanwhile, conducted a new exploration in LINE-1 inhibitors by combining its regulation mechanisms.

**FIGURE 1:**
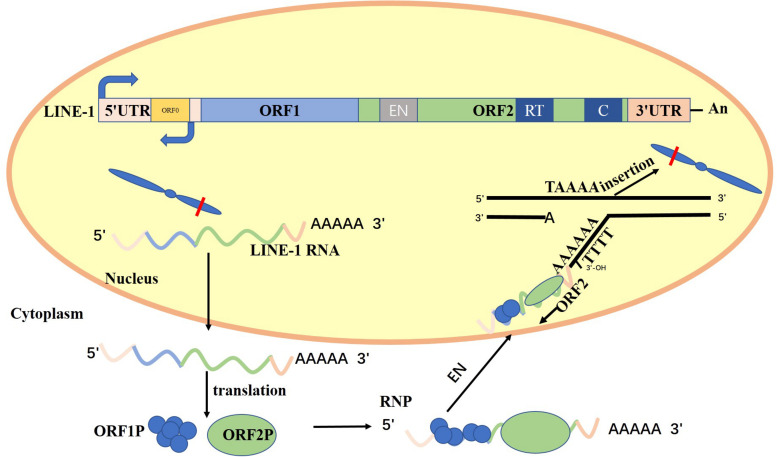
Structure of LINE-1 and LINE-1 retrotransposition cycle.

Figure 1 shows the relative positions of the 5′ untranslated region (5′-UTR); the open reading frames ORF0, ORF1, and ORF2; the 3′ untranslated region (3′-UTR); and the Poly A tail. ORF2 encodes endonuclease (EN), reverse transcriptase (RT), and cysteine-rich domain (C). Full-length LINE-1 mRNA was generated using the sense promoter at 5′-UTR. The LINE-1 mRNA is exported to the cytoplasm where ORF1 and ORF2 are translated and combined to form a ribonucleoprotein (RNP) particle. The RNP is then incorporated into the nucleus, and the ORF2P endonuclease in the RNP identifies and cuts specific sequences on the bottom DNA strand at the consensus site 3-′AA/TTTT−5′. Subsequently, the free 3′ hydroxyl generated at the fracture is utilized by the ORF2P and LINE-1 mRNA in the RNP is used as the template for reverse transcription to produce the complementary DNA of the LINE-1 gene (Richardson et al., 2015; Kazazian and Moran, 2017).

## Line-1 and Disease

### LINE-1 and Cancer

When LINE-1 retrotransposition is out of control, it can lead to diseases. More than 1,000 articles focusing on LINE-1 and cancer are available in the PubMed archive (Rodic, 2018).

### LINE-1 Hypomethylation and Cancer

The global hypomethylation of the genome promotes chromosomal instability, genomic instability, and genetic heterogeneity because specific changes in DNA methylation can affect a variety of genome sequences, especially the intergenic and intronic regions of the DNA, resulting in chromosome instability and mutations (Wilson et al., 2007). LINE-1 promoter hypomethylation is a biomarker for genome-wide DNA hypomethylation, which is itself a major hallmark of cancer. Thayer et al. (1993) first demonstrated the methylation status of LINE-1 in cancer cells. Since then, LINE-1 hypomethylation of tumors has attracted widespread attention (Thayer et al., 1993). LINE-1 hypomethylation was reported to be associated with poor survival in more than 200 cases of gastric cancer, suggesting its potential as a prognostic biomarker (Shigaki et al., 2013). This phenomenon was also subsequently found in lung cancer, liver cancer, esophageal cancer, prostate cancer, and endometrial cancer (Iwagami et al., 2013; Kawano et al., 2014; Lavasanifar et al., 2019). Ogino et al. (2008) analyzed 643 colon cancer samples from two independent prospective cohorts, demonstrating a linear correlation between LINE-1 hypomethylation and aggressive tumor behavior. It has been reported that global DNA hypomethylation promotes aggressive tumor behavior by amplifying oncogenes or through abnormal expression of microRNAs (Baba et al., 2014, 2018). In esophageal cancer with high mortality and poor endoscopic screening sensitivity, LINE-1 hypomethylation can serve as a good diagnostic biomarker, thereby improving 5-year survival (Shah et al., 2013). LINE-1 hypomethylation can also be seen in some precancerous lesions. For example, in colorectal cancer, LINE-1 hypomethylation had no significant difference between adenomas and cancerous tissues, but it was significantly lower in adenomas than in normal tissues (Dawwas, 2014). Therefore, LINE-1 hypomethylation also can be used as an early biomarker for cancer.

However, there was no significant difference in the hypomethylation of LINE-1 between the blood samples of patients with leukemia and those of normal subjects (Barchitta et al., 2014).

### LINE-1 Integrations and Cancer

Many tumor tissues have been found to present a high level of LINE-1 activity that can rapidly increase their copy number through the “copy-and-paste” mechanism (Dunaeva et al., 2018). LINE-1 can be used as cis-regulatory elements to regulate the expression of host genes (Wanichnopparat et al., 2013). Pan-cancer Analysis of Whole Genomes analysis of 2,954 cancer genomes from 38 histological subtypes revealed that aberrant LINE-1 integrations could lead to gene rearrangement (Rodriguez-Martin et al., 2020). LINE-1-mediated rearrangement can trigger oncogene amplification. In breast cancer, Morse and colleagues first proposed that hypomethylation activates LINE-1 which can utilize the target primed reverse transcription pathway to insert into the oncogene MYC, causing tumor-specific rearrangement and amplification (Morse et al., 1988). LINE-1 was found to induce the amplification of CCND1 oncogene in esophageal tumor by inducing the breakage–fusion–bridge cycles (Rodriguez-Martin et al., 2020). LINE-1 can mediate the deletion of tumor suppressor genes. It may be through X inactivation mechanism that LINE-1 mRNA forms facultative heterochromatin in the inactivated region or LINE-1 mRNA and pre-mRNA form RISC complex to degrade complementary mRNA (Allen et al., 2003; Aporntewan et al., 2011). In colon cancer, Miki et al. reported that LINE-1 insertion disrupts the tumor suppressor gene APC, which can lead to gene inactivation (Miki et al., 1992). In lung squamous cell carcinoma, we found that LINE-1 insertion into tumor suppressor gene FGGY promotes cell proliferation and invasion *in vitro*, and facilitates tumorigenesis *in vivo* (Zhang et al., 2019).

### High Expression of ORF1 and ORF2 of LINE-1 and Cancer

The activation of LINE-1 increases the translation of ORF1 and ORF2, which are not expressed in normal somatic tissues. ORF1 encodes an RNA-binding protein, and high expression level of ORF1 was proved to be more common in most of the cancers and therefore as a diagnostic marker. In breast cancer, high expression of nuclear ORF1 is associated with distant metastasis and poor prognosis (Harris et al., 2010). In high-grade ovarian carcinoma, the ORF1 level was high and correlated to the loss of TP53 (Rodic et al., 2014). The expression of both the LINE-1 ORF1 and c-Met protein was significantly increased and peaked in early stage in ovarian cancer, suggesting that LINE-1 ORF1 significantly activates c-Met (Ko et al., 2019). In tumor cell experiments, increased mRNA and protein expression of LINE1-ORF1 can result in significant enhancement in cell proliferation and colony formation (Tang et al., 2018). It is worth noting that the expression of ORF1 was heterogeneous and had histological specificity. Cancers originating in the endometrium, such as biliary tract, esophagus, bladder, head and neck, lung, and colon, exhibit ORF1 overexpression, whereas other cancers, such as renal, liver, and cervical cancer, show little expression of ORF1 (Ardeljan et al., 2017). Recent studies have shown that an ELISA method to measure ORF1 in serum can be better in prostate cancer detection (Hosseinnejad et al., 2018).

ORF2 encodes a protein with reverse transcriptase and endonuclease activities. High expression of endonuclease induces double-strand DNA breakage that can aggravate DNA damage repair and increase genomic instability (Kines et al., 2014). Reverse transcriptase activation can promote cell proliferation and differentiation and also alter the non-coding RNA transcription spectrum and other epigenetic phenotypes, resulting in alterations in cell regulatory networks, tumor development, and other important pathological processes (Rodic and Burns, 2013; Burns, 2017; Christian et al., 2017). ORF2 can express early in the tumorigenesis process, as it can be detected by a highly specific monoclonal antibody (mAb chA1-L1) in both transitional colon mucosa and prostate intraepithelial neoplasias (De Luca et al., 2016). However, studies have shown that chA1-L1 recognizes both ORF2p and the transcriptional regulator BCLAF1, so it is not specific (Briggs et al., 2019). But recently, tumor proteome profiling studies based on mass spectrometry have shown that ORF2p was difficult to be detected, and after affinity capture of ORF1p, ORF2p has not been detected in stem cell LINE-1 proteome analysis (Vuong et al., 2019; Ardeljan et al., 2020). Therefore, the detection and application of ORF2 in tumors are still worth exploring.

### LINE-1 and Metabolic Disorders

New research has shown that LINE-1 is also associated with blood sugar and lipid levels (Turcot et al., 2012). LINE-1 methylation is associated with type 2 diabetes mellitus (T2DM). Studies showed that, compared with hypermethylation, LINE-1 hypomethylation was associated with a higher risk of worsening metabolic status, independent of other classic risk factors (Martin-Nunez et al., 2014). This discovery highlights the potential role for LINE-1 DNA methylation as a predictor of the risk of T2DM or other related metabolic disorders. LINE-1 DNA methylation is associated with increased LDL cholesterol and decreased HDL cholesterol levels, and these metabolic changes increase the risk of cardiovascular disease (Pearce et al., 2012). LINE-1 DNA methylation is also associated with many blood-based metabolic biomarkers. In fetal neural tissue with neural tube defects, it was found that the low methylation level of LINE-1 was associated with the significant reduction of vitamin B_12_ in maternal plasma, as well as lower folate levels and increased concentrations of homocysteine (Wang et al., 2010). Folic acid and other B vitamins play an important role in the biosynthesis of new purines and pyrimidines. Therefore, the methylation status of LINE-1 can be a predictor of some metabolic diseases. Current studies have shown that LINE-1 can also regulate metabolism by inserting metabolic genes. It was reported that LINE-1 insertions in the FGGY gene can upregulate cytochrome P450, arachidonic acid metabolism, and glycerolipid metabolism. These metabolic disorders can lead to the occurrence of a variety of diseases and poor prognosis (Zhang et al., 2019).

### LINE-1 and Neurological Disorders

LINE-1 can affect the developing brain at different stages of health and disease (Suarez et al., 2018). Ataxia telangiectasia (AT) is a progressive neurodegenerative disease caused by ataxia telangiectasia mutated (ATM) gene mutation. In 2011, researchers found that in nasopharyngeal carcinomas with ATM deficiency, LINE-1 retrotransposition increased, and ORF2 copy number increased in AT neurons, thus verifying the correlation between LINE-1 retrotransposition and ATM deficiency (Coufal et al., 2011). High expression of LINE-1 was found in Rett syndrome caused by mutation of methyl CpG binding protein 2 (MeCP2) in the X-linked gene, which was caused by the inclusion of LINE-1 5′-UTR sequence in the MeCP2 target, leading to methylation-dependent repression (Muotri et al., 2010). LINE-1 is involved in the aging process. In patients with frontotemporal lobe degeneration, LINE-1 transcripts were found to be elevated (Li et al., 2012). LINE-1 hypomethylation has been observed in most psychiatric studies. Increased copy numbers of LINE-1 as a result of LINE-1 hypomethylation were also found in patients with schizophrenia, bipolar disorder, and major depressive disorder (Liu et al., 2016; Li et al., 2018). The link between LINE-1 methylation levels and Alzheimer’s disease is still being studied.

### LINE-1 and Genetic Disorders

LINE-1 is reported to be related to chromosome disorders. The first observation of LINE-1 insertion was in 1988, when Kazazian et al. observed a new exon of F8 LINE-1 insertion in the X-linked gene, which is a gene encoding coagulation factor VIII in a patient with hemophilia A (Kazazian et al., 1988). Then, a LINE-1 insertion was found in the CHM gene of a patient diagnosed with choroideremia. The reverse integration of a LINE-1 element into exon 6 resulted in aberrant splicing of the CHM mRNA (van den Hurk et al., 2003). Furthermore, LINE-1 can also promote mobilization of other RNAs in trans, Alu, and SVA, which can be trans-mobilized, leading to gene insertions (Kemp and Longworth, 2015). Retrotransposon insertions were found to account for up to 0.4% of all NF1 mutations (Wimmer et al., 2011). Neurofibromatosis type I is an autosomal dominant disorder caused by NF1 gene mutations (Messiaen et al., 2011). Alu insertion is located 44 bp upstream of NF1 exon 41, causing the exon 41 to skip and change the open reading frame (Payer and Burns, 2019). Only two cases were thought to be a result of independent SVA insertion in SUZ12P accompanied by 867-kb and 1-Mb deletions that encompassed the NF1 gene (Vogt et al., 2014). In autosomal recessive genetic disease, such as Fanconi anemia caused by SLX4^FANCP^ deficiency and Aicardi–Goutieres syndrome (AGS) of three-prime repair exonuclease 1 mutations, LINE-1 expression was upregulated and pro-inflammatory cytokines were produced through the cGAS–STING pathway (Brégnard et al., 2016; Suarez et al., 2018).

## Line-1 and Immune Regulation of Disease

### LINE-1 and Autoimmune Disease

Hypomethylated and highly expressed LINE-1 has been found in autoimmune diseases such as systemic lupus erythematosus (SLE), Sjögren’s syndrome (SS), and psoriasis (Schulz et al., 2006; Yooyongsatit et al., 2015; Mavragani et al., 2016). LINE-1 RNA is characterized by viral RNA and exists as RNP particles, which can be recognized by RNA sensors and activate innate immune responses (Mavragani et al., 2016). Cell studies demonstrated that LINE-1 activates the production of IFNβ by RNA pathway (Zhao et al., 2018). When LINE-1 retrotransposition was inhibited by RT inhibitors, significant reductions were observed in IFNα, IFNβ, and IFNγ mRNA levels (Brégnard et al., 2016). LINE-1 transcripts and p40 protein (a 40−kDa RNA binding protein) that LINE-1 encodes have been detected in SLE and SS patients. It has been demonstrated that LINE-1 can induce the production of IFN-I *in vitro* by TLR-dependent and TLR-independent pathways (Mavragani et al., 2016). In MRL autoimmune lymphoproliferative syndrome, LINE-1 ORF2 encoding an RT and its products are associated with an MHC class I molecule on the cell membrane (Benihoud et al., 2002). In Fanconi anemia and AGS, LINE-1 was found to be associated with the activation of the autoimmune system. LINE-1 also regulates immunity by acting as a cis-regulatory element through the mechanism of LINE-1 mRNA and pre-mRNA forming RISC complex to degrade the complementary mRNA (Wanichnopparat et al., 2013).

### LINE-1 and Tumor Immunity

In 112 TCGA cancer samples, the scientists measured the transcriptional activity of 1789 pathways and found that 49 of 176 immune pathways were significantly negatively correlated with LINE-1 (Jung et al., 2018). LINE-1 is inversely correlated with the expression of immunologic response genes. Less LINE-1 activity was found in tumors with high immune activity. In esophageal cancer tissues, scientists found that the LINE-1 methylation level in tumors was significantly positively associated with the peritumoral lymphocytic reaction (Kosumi et al., 2019). The activities of regulatory T cells and PD1 signaling as reported in cancer immune evasion and chronic inflammatory conditions also have negative correlations with LINE-1. It is reported that the negative correlation between LINE-1 and immune activity may be caused by the destruction of LINE-1 inhibition, but the specific mechanism is still unclear. LINE-1 may also mediate immune tolerance, which may change from immune stimulation mode to immunosuppression mode through continuous IFN signaling or directly affect lymphocyte signaling.

### LINE-1 and Metabolism-Induced Immunity

LINE-1 is also associated with blood sugar and lipid levels. Abnormal glucose and lipid metabolism can lead to metabolic reprogramming in tumor cells. The most classic metabolism of tumor is Warburg effect, where a large amount of glucose is absorbed to fulfill the need for proliferation and produce lactic acid (Lunt and Vander Heiden, 2011). The acidic microenvironment caused by lactic acid leads to impaired T-cell activation and proliferation, prevents NK cell activation, stabilizes HIF1α to stimulate the polarization of anti-inflammatory M2 macrophages, and inhibits the production of IFN-γ in tumor-infiltrating T cells (Husain et al., 2013; Colegio et al., 2014; Brand et al., 2016). Abnormal lipid metabolism in tumor cells also can lead to local immunosuppression in the microenvironment (Hao et al., 2019). LINE-1 can affect local immune homeostasis by inserting elements into metabolism-related genes. FGGY is known to encode a protein that phosphorylates carbohydrates and is associated with obesity and sporadic amyotrophic lateral sclerosis (Zhang et al., 2011). LINE-1 retrotransposons suppress FGGY, leading to lipid metabolism disturbance and diet-induced obesity in mice (Taylor et al., 2018). Lung squamous cell carcinoma patients with L1-FGGY^+^ tissue have a poor prognosis, have low levels of CD3^+^ T cells, and have high levels of CD68^+^ macrophages and CD33^+^ myeloid-derived cells (Zhang et al., 2019). L1-FGGY^+^ also regulates the abnormal transcription of cytokines related to the immunosuppressive micromilieu.

## Line-1 Inhibition

**FIGURE 2:**
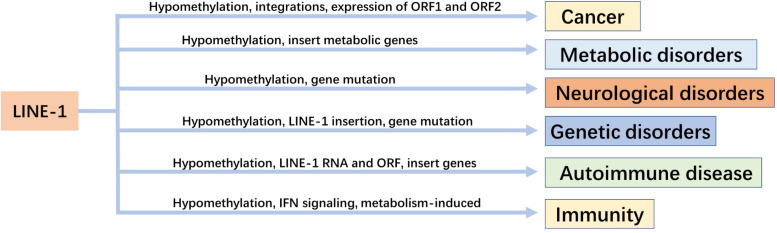
The relationship between LINE-1 and diseases and their regulatory mechanisms.

The correlation between LINE-1 and disease as well as immunity was analyzed (Figure 2). The life cycle of LINE-1 provides a plethora of ways to target and inhibit LINE-1 expression (Banuelos-Sanchez et al., 2019). The inhibition of LINE-1 has become a treatment strategy for some diseases.

### Targeting LINE-1 Methylation

Full-length LINE-1 transcription is driven by a CpG dinucleotide-rich internal promoter. Hypomethylation of LINE-1 causes the activation of LINE-1, which causes retroelement transposition and chromosomal alteration (Saito et al., 2010). The hypomethylation of LINE-1 has become an important factor in the occurrence and development of diseases, so maintaining the state of LINE-1 methylation has become a key method for the treatment of diseases. Soy isoflavone supplementation can regulate the level of LINE-1 methylation in head and neck squamous cell carcinoma (HNSCC). In a clinical trial of 39 patients with HNSCC who took a soy isoflavone supplement (300 mg/day) orally for 3 weeks before surgery, a positive correlation was found between LINE-1 methylation level and daily isoflavone intake (Rozek et al., 2019). Some cell-based studies and clinical data have shown that LINE-1 dysregulation is associated with tumor drug resistance (Zhu et al., 2015; Lavasanifar et al., 2019). It was found in breast cancer cells treated with paclitaxel that DNMT3a, a member of the DNA methyltransferase family, could enhance the methylation level in the gene by binding to the inner region of the LINE-1 gene, and then upregulate the expression level of LINE-1. Downregulating the expression of DNMT3a can effectively inhibit the expression of LINE-1 (Wang et al., 2020). LINE-1 retrotransposon silenced also through histone modifications. Histone demethylase KDM4B may enhance the LINE-1 retrotransposition efficacy, whereas depletion of KDM4B reduced it in breast cancer (Xiang et al., 2019). Elevated LINE-1 expression was found in PC9 drug-tolerant persister (DTP) cancer cells treated with the EGFR inhibitor erlotinib. HDAC inhibitors can suppress LINE-1 in DTP cancer cells (Guler et al., 2017). Currently, DNA methyltransferase inhibitors and histone deacetylase inhibitors have entered clinical trials (Gaillard et al., 2019).

### Targeting RT Activity

LINE-1 elements harbor ORF1 and ORF2, which has reverse transcriptase (RT) activity, and RT inhibition may be a novel, non-cytotoxic anticancer therapy strategy (Sciamanna et al., 2018). RT is a key player in retrotransposition and functions by transcribing LINE-1 mRNA or other RNAs to cDNA at the integration sites (Khalid et al., 2018). Specific reverse transcription inhibitors, including nevirapine (NVR) and efavirenz (EFV), which target the HIV-1-encoded RT and are currently used in AIDS therapy, reduce cell proliferation and promotes differentiation of a variety of cancer cell lines of unrelated histological origin (Mangiacasale et al., 2003; Landriscina et al., 2005; Sciamanna et al., 2005). *In vivo* assays using murine models inoculated with various human cancer cell lines revealed that daily treatment of animals with EFV significantly delayed the progression of tumors (Oricchio et al., 2007). NVR and EFV dramatically countered L1-FGGY abundance, inhibited tumor growth, attenuated metabolism dysfunction, and improved the local immune evasion in lung squamous cell carcinomas (Zhang et al., 2019). EFV has recently undergone a phase II clinical trial in patients with metastatic prostate cancer (Houédé et al., 2014). Another RT inhibitor, F2-DABOs, has shown anti-proliferative activity in nude mice, helping to promote cell differentiation and inhibit tumor growth (Sbardella et al., 2011). Later, the nucleoside reverse transcription inhibitor abacavir was also shown to inhibit cell growth, migration, and invasion (Carlini et al., 2010). Capsaicin is the main chemical component of *Asiasari radix* and *Capsicum annuum*, as well as the major component of a Chinese traditional herbal medicine, Sho-seiryu-to (Friedman et al., 2018). Capsaicin suppresses LINE-1 by inhibiting the RT activity of LINE-1 ORF2P, which is the LINE-1-encoded RT responsible for LINE-1 activity (Nishikawa et al., 2018). A recent study revealed that azidothymidine (AZT) inhibits the RT activity of ORF2P in a fetal oocyte attrition model. Experiments showed that AZT-treated oocytes have a reduction of LINE-1 ORF1 ssDNA compared with untreated oocytes (Tharp et al., 2020). It is important to note that RT inhibitors do not eliminate the tumor but only control its progression. Therefore, in addition to the anti-AIDS drugs approved by the FDA, the combination of Chinese and western medicine can be regarded as an emerging treatment.

### Combined Immunotherapy

Recent studies suggest that LINE-1 hypomethylation may be a positive indicator of immunotherapy. DNA methyltransferase (DNMT) is an important epigenetic molecule that catalyzed DNA methylation and can induce the development of various tumors. Downregulating the expression of DNMT can effectively inhibit the expression of LINE-1 (Wang et al., 2020). So DNA methyltransferase inhibitors (DNMTis) play an important role in the anti-tumor process. DNMTI can improve tumor immunogenicity, promote NK cells and CD8^+^ T cells to play a cell-mediated cytotoxic role, and promote immune response to participate in antigen commission by regulating immunosuppressive cells (Chiappinelli et al., 2015). DNMTi can enhance the expression of cancer-testis (CT) antigen, making the tumor more susceptible to CT antigen vaccine. The combination of decitabine, a DNA methyltransferase inhibitor, and cancer-testis/cancer-germline antigen NY-ESO-1 vaccine has a good therapeutic effect in the primary treatment of human recurrent epithelial ovarian cancer (Odunsi et al., 2014). A clinical trial has shown that combination therapy with carboplatin and anti-programmed death-1 has a good therapeutic effect in lung cancer because carboplatin can induce LINE-1 expression (Langer et al., 2016). Therefore, LINE-1 can be used as a target of combined immunotherapy in tumor therapy.

### Other Inhibitors

Recently, a number of other regulatory approaches have been reported. In somatic cells, microRNAs (miRNAs or miRs) also regulate the activity of LINE-1 (Idica et al., 2017). MiR-128 regulates LINE-1 activity in somatic cells by targeting the nuclear import factor transportin-1 (TNPO1) 3′-UTR, which mediates nuclear import and requires RanGTP for cargo delivery into the nucleus (Twyffels et al., 2014). MiR-128 inhibits the expression of TNPO1 mRNA and protein, and TNPO1 deficiency suppresses LINE-1 mobilization by inhibiting nuclear import of LINE-1–RNP (Idica et al., 2017). MiR-128 also guides the miRNA-induced silencing complex to bind directly to a target site residing in the ORF2 RNA of LINE-1 (Hamdorf et al., 2015). At present, a novel target of miR-128 has been identified as heterogeneous nuclear ribonucleoprotein A1 (hnRNPA1), which is required for LINE-1 retrotransposition (Goodier et al., 2013; Fung et al., 2019). MiR-128 represses hnRNPA1 mRNA and protein by targeting the CDS of hnRNPA1, which interacts with LINE-1 ORF1p via RNA bridge to promote LINE-1 mobilization (Goodier et al., 2013). This interaction results in translational repression of the LINE-1 retrotransposition, thereby reducing the risk of LINE-1-mediated mutagenesis (Pedersen and Zisoulis, 2016). Therefore, microRNAs can be a target for LINE-1 inhibition.

Aryl hydrocarbon receptor (AHR) is a ligand-activated transcription factor that activates LINE-1 expression (Teneng et al., 2007). AHR is overexpressed in breast and thyroid cancers, suggesting that these tumors also overexpress LINE-1 (Powell et al., 2013). Lai et al. found that biseugenol, a novel AHR inhibitor, impeded cancer growth and inhibited EMT in gastric cancer cells (Lai et al., 2014). These findings suggest that targeting AHR with small molecule inhibitors may be a novel therapeutic approach. ORF1P phosphorylation by protein kinase A is also required for LINE-1. Kinase inhibitors specifically designed to target LINE-1 ORF1P phosphorylation may be associated with inhibition of LINE-1 (Bojang et al., 2013). Therefore, there is room for drug development research focusing on targeting and inhibiting LINE-1 ORF1P phosphorylation.

## Conclusion

The activation of LINE-1 retrotransposon is associated with a variety of human diseases and is involved in the occurrence and progression of disease through retrotransposition-dependent and retrotransposition-independent mechanisms. Currently, it has even become a marker of tumorigenesis and prognosis and is related to immune regulation. The effective inhibition of LINE-1 activation has become a treatment for some diseases. The inhibition of LINE-1 in animal experiments can inhibit the occurrence and development of tumors, so the clinical application of LINE-1 inhibitors is imminent. In addition to exploring some known inhibitors, other mechanisms of LINE-1 inhibition should also be explored. We summarized the relationship between LINE-1 and disease-related immunity, and proposed that LINE-1 may affect the immune status of the body by regulating metabolism, leading to poor prognosis. Metabolic substances can affect the immune microenvironment, for example, lactic acid can lead to immunosuppressive microenvironment, leading to poor prognosis of tumors. The dysregulation of LINE-1 can lead to the disorder of glucose and lipid metabolism, and the inhibition of glucose and lipid metabolism may reverse the disease progression caused by LINE-1. Now the anti-tumor effect of regulating the body’s metabolism has entered clinical trials, such as the significant effect of metformin in the treatment of tumors. Therefore, the metabolic status of diseases caused by LINE-1 can be checked. Metabolic therapy combined with LINE-1 inhibitors may inhibit the progression of LINE-1 and may improve the immune microenvironment to achieve the optimal therapeutic effect.

## Author Contributions

XZ wrote the article. RZ and JY reviewed and revised the article. All authors contributed to the article and approved the submitted version.

## Conflict of Interest

The authors declare that the research was conducted in the absence of any commercial or financial relationships that could be construed as a potential conflict of interest.
